# Effect of Salinity Stress on Growth, Physiology and Anatomy of Pumpkin (*Cucurbita moschata* Duchesne) cv. Kang Kog

**DOI:** 10.21315/tlsr2025.36.2.7

**Published:** 2025-07-31

**Authors:** Chaichan Maneerattanarungroj, Sununta Sudjai, Narisa Kunpratum, Pitakpong Maneerattanarungroj, Worasitikulya Taratima

**Affiliations:** 1 Department of Biology, Faculty of Science, Naresuan University, Phitsanulok 65000, Thailand; 2 Department of Biology, Faculty of Science, Khon Kaen University, Khon Kaen 40002, Thailand; 3 Faculty of Veterinary Medicine, Khon Kaen University, Khon Kaen 40002, Thailand

**Keywords:** Anatomical Adaptation, Growth, NaCl, Pumpkin, Salinity Stress

## Abstract

Pumpkin is an important economic crop with high nutritional value. Different pumpkin varieties experience diverse growth problems due to soil salinity. This research studied the physiological and anatomical adaptations of the Kang Kog pumpkin cultivar to salinity stress. Pumpkin seedlings were grown under a hydroponic system using Hoagland’s solution with NaCl concentrations of 0 mM, 25 mM, 50 mM, 75 mM and 100 mM for four weeks. Results showed that pumpkin leaf number, leaf width, leaf length, root number, root length, plant height, stem diameter, fresh weight and dry weight significantly decreased after exposure to high NaCl concentrations. Chlorophyll a and green intensity measured as SPAD units also significantly decreased, while chlorophyll fluorescence (Fv/Fm, Fv’/Fm’) and chlorophyll *b* content of all treated groups were not significantly different when compared to the control group. Fibre strands and cuticles in all treatments were significantly thicker compared to the control group, while vessel diameters and vascular bundle sizes of the treated groups significantly decreased compared to the control group. Results showed that salinity stress did not impact chlorophyll *b* and chlorophyll fluorescence. Kang Kog pumpkins can adapt and grow in slightly saline environments. Our results provide important information for pumpkin breeding programs efforts that can be used in combining with other agronomic characters to improve tolerant cultivars under initial salinity stress tolerance.

**Highlights**

- High salinity stress inhibited the growth of Kang Kog pumpkin, whereas low salinity stress had minimal impact on its development.
- Chlorophyll fluorescence and chlorophyll *b* content were not significantly affected by salinity stress.
- Fiber strand thickness and cuticle thickness were identified as key anatomical traits for assessing and understanding salt tolerance in pumpkins.

## INTRODUCTION

Soil salinity refers to the presence of excessive amounts of soluble salts in the soil that negatively impact agricultural productivity, leading to low economic returns (Hu & Schmidhalter 2002). Salinity stress reduces plant growth and development by adversely impacting physiological processes through increased intracellular osmotic pressure. This leads to the accumulation of sodium at toxic levels (Zhao *et al*. 2021), which negatively affects plant growth and reproduction (Shrivastava & Kumar 2015) by producing nutritional and hormonal imbalances (Blaylock 1994), ion toxicity (Shrivastava & Kumar 2015), oxidative and osmotic stress (Qari *et al*. 2022; Mustafa *et al*. 2023), and increased susceptibility to diseases (Mustapha *et al*. 2022). High salt concentration alters soil porosity and hydraulic conductivity, leading to low soil water potential and physiological drought conditions (Mehnert & Jennings 1985; van Dijk *et al*. 2017). Plants respond to salinity stress by accumulating compatible solutes, redistributing ions and increasing endogenous abscisic acid (ABA) content. Under saline stress conditions, plants exhibit physiological and biochemical adaptations by producing antioxidant chemicals, suitable solute synthesis and ion homeostasis. Previous studies reported that plant adaptability and salt stress tolerance are significantly influenced by microtubules (Chun *et al*. 2021), plant growth-promoting bacteria (Shilev 2020), phytohormones (Kaya *et al*. 2009; Anjum *et al*. 2023), and some organic acids (Yang *et al*. 2023). Crop species vary in their response to salinity stress, with some showing significant reductions in yield components and overall yield.

Pumpkin is considered a healthy vegetable that is rich in fibre, vitamins, minerals and antioxidants (Aziz *et al*. 2023). Salt stress has significant negative impacts on pumpkin growth. Previous studies have shown that salt stress affects pumpkin and squash growth including germination, seedling development, and overall plant growth (Kurum *et al*. 2013; Dantas *et al*. 2019; Opande & Khasabulli 2022; Taratima *et al*. 2022; 2023; Tarchoun *et al*. 2022; Irik & Bikmaz 2024). Seedling growth was inhibited at all salt stress levels, with root length more severely inhibited than shoot length. Different salt concentrations affect the malondialdehyde (MDA) content, free proline and chlorophylls in pumpkin and squash seedlings, with oxidative stress and damage to cellular structures leading to increased evapotranspiration which adversely impacts plant growth and fruit setting (Sevengor *et al*. 2011).

In Thailand, the inland salt-affected soils are formed by geochemical processes and cover approximately 1.841 million ha in the northeastern, whereas coastal salt-affected soils are formed from seawater scattered along the coast of 0.425 million ha, while 0.063 million ha are found in other regions. Typically, salinity soils are categorised as slightly, moderately, severely salt-affected soils and potential salt source area. This classification aligns with the management practices (Arunin & Pongwichian 2015). Nowadays, the improvement of saline soils continues to be carried out, including the selection of appropriate plant species suitable for growing in saline soil areas. One of the interesting plants is pumpkin due to its deep roots, which facilitate the absorption of groundwater (Bischoff 2018). However, information on how various local Thai pumpkin cultivars respond to different salinity levels is still deficient. To the best of our knowledge, there are no publications on the physiology and anatomical traits of pumpkin (*Cucurbita moschata* Duchesne.) cv. Kang Kog, the most popular pumpkin variety grown in Thailand, in relation to salinity stress. Therefore, this research investigated the physiological and anatomical responses of *C. moschata* cv. Kang Kog to different salinity stress levels under hydroponic conditions. The results will enhance understanding and support the improvement of pumpkin cultivation programmes under various salinity levels.

## MATERIALS AND METHODS

### Plant Materials and Culture Conditions

Seeds of *Cucurbita moschata* cv Kang Kog were germinated separately in 3 cm × 5 cm soil pots with daily watering for 14 days. Following germination, seedlings with one to two leaves were placed in 10 cm by 25 cm rectangular tubes with a foam sheet as explants for hydroponic cultivation using the Deep Flow Technique (DFT). One liter of Hoagland’s solution (pH 5.5) was added to each tube, and the tubes were then aerated with an air pump (model ACQ-007, 75 watts, 100 l/s). The pumpkin seedlings were cultivated for four weeks at 27 ± 2°C under 40 μmol/m^2^/s light intensity in Hoagland’s solution containing 0 as the control, 25 mM, 50 mM, 75 mM and 100 mM NaCl. Plant height, biomass, stem diameter, healthy roots longer than 2 cm, leaves longer than 2 cm and leaf size were recorded.

### Green Intensity and Chlorophyll Content

A SPAD-502 Plus Chlorophyll Meter was used to measure the amount of green leaf at the base, middle and apex of mature leaf blades in SPAD units. Total chlorophyll, chlorophyll *a* and chlorophyll *b* were used to measure the amount of chlorophyll present in mature leaves. Five milliliters of 80% acetone were added to 0.1 g of mature leaves and ground in a mortar until all the green material had dissolved. Then, 20 mL of 80% acetone was added and the mixture was filtered through filter paper. A spectrophotometer (Spectronic 20) was utilised to measure the absorbance values at 645 nm and 663 nm, with 80% acetone serving as the blank. The formulae below were applied to determine the chlorophyll contents following Arnon (1949).


$$\begin{array}{l} \text{Total chlorophyll }(\text{mg}/\text{g tissue})=20.2\;(\text{A}645)+8.02\;(\text{A}663)\times \text{V}/(1,000\times \text{W})\\ \text{Chlorophyll }a\;(\text{mg}/\text{g tissue})=12.7\;(\text{A}663)-2.69\;(\text{A}645)\times \text{V}/(1,000\times \text{W})\\ \text{Chlorophyll }b\;(\text{mg}/\text{g tissue})=22.9\;(\text{A}645)-4.68\;(\text{A}663)\times \text{V}/(1,000\times \text{W}) \end{array}$$

where, V = total volume of solution (mL) and W = weight of leaves (g).

Mature leaves were measured using a Chlorophyll Fluorometer Handy PEA as dark-adapted leaves (30 min dark) (Fv/Fm units) and light condition (Fv’/Fm’ units) to examine chlorophyll fluorescence (Theerakulpisut 2016), with each measurement repeated three times.

### Anatomical Investigation

Mature pumpkin stems were cut at 1 cm–3 cm from the root, fixed in 100 mL of FAA70 fixative (70% ethyl alcohol, acetic acid and formaldehyde; 90:5:5) (Johansen 1940), and used as explants in this anatomical study. A free-hand method was used to section the explants transversely. The samples were first stained with 1% (w/v) Safranin O solution before dehydrating using an ethyl alcohol and xylene series and then mounted using DePeX. Anatomical features such as fibre strand thickness, cuticle thickness, vascular bundle size and vessel size were recorded using a light compound microscope (ZEISS Axiocam ERc 5s, country). The Axio Vision LE64 application was then used to score these features following Taratima *et al*. (2019).

### Statistical Analysis

Each treatment was conducted in triplicate using a completely randomised design (CRD). One-way analysis of variance (one-way ANOVA) was used to verify the statistical analyses, with the post hoc test (Duncan’s test) at 95% confidence level used to compare mean values. Principal component analysis (PCA) and Pearson correlation analysis were accomplished using Origin 2022 software to examine the growth and anatomical response of Kang Kog pumpkin plants.

## RESULTS

**Figure 1:**
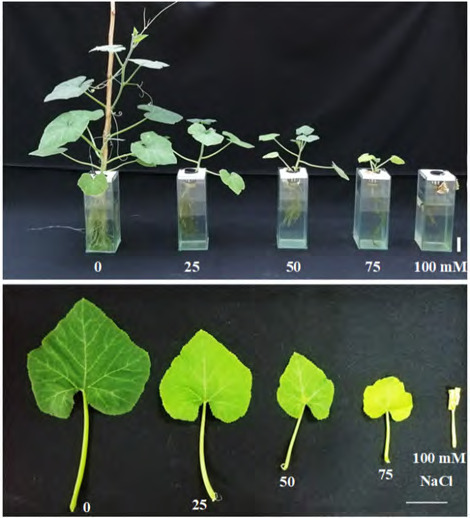
Growth and leaf features of Kang Kog pumpkin grown under different salinity stress levels for four weeks. Scale = 5 cm.

After four weeks of NaCl treatment, the growth of pumpkin seedlings including plant height, root number and length, leaf number and size, and fresh and dry weight reduced as NaCl concentration increased (Table 1, Fig. 1). The leaves turned pale-yellow after two weeks of treatment. Leaf size and number decreased compared to the control group. In the 100 mM NaCl group, the leaves withered in the third week and completely dried in the fourth week. Therefore, chlorophyll content and green intensity values were not reported in this treatment. Increased salinity also resulted in a decrease in root number. All the experimental groups showed a statistically significant reduction in root number compared to the control group.

Salinity stress impacted the chlorophyll content in Kang Kog pumpkin leaves. Chlorophyll *b* content decreased in all treatments but were not significantly different (Table 1), while chlorophyll *a* content and green intensity (SPAD units) of the 50 mM and 75 mM NaCl treatment groups were significantly lower than the control group. Chlorophyll fluorescence measured in dark-adapted leaves and light condition decreased with increasing sodium chloride concentrations but were not significantly different.

**Figure 2:**
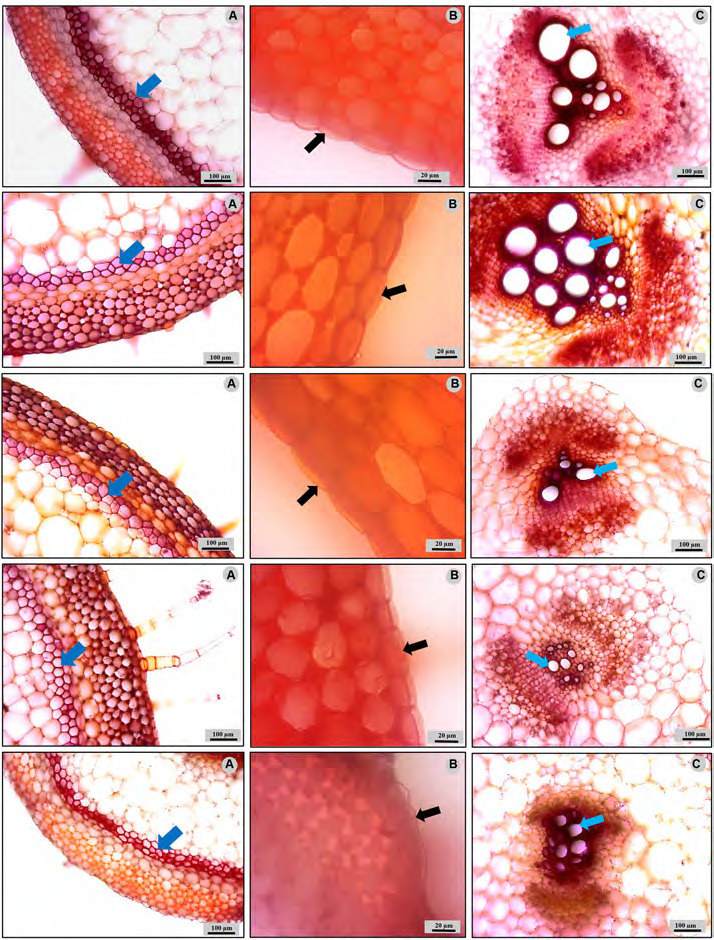
Anatomical characteristics of pumpkin stems grown under salinity stress. (A) Fibre strand in cortex region (arrows); (B) cuticle thickness (arrows) and (C) vessel in vascular bundle (arrows).

Stem anatomical analysis showed that fiber strand thickness in the cortex significantly enlarged when NaCl concentration increased, except for the 100 mM NaCl group. The highest fiber strand thickness was found in plants from the 75 mM NaCl group, while the lowest was recorded in the control group (Table 1, Fig. 2A). The cuticle thickness increased with increasing concentrations of NaCl, with statistical significance. In all groups, cuticle thickness was significantly lower than the 75 mM NaCl group, except for the 100 mM concentration group (Table 1, Fig. 2B). Vascular bundle sizes of all treatments dramatically decreased when NaCl concentration increased, except for the vascular bundle length of the 25 mM NaCl group. Vessel diameter significantly decreased with increasing NaCl concentration. The highest vessel diameter was measured in the control group, while the smallest was found in the 100 mM NaCl group (Table 1, Fig. 2C).

**Figure 3:**
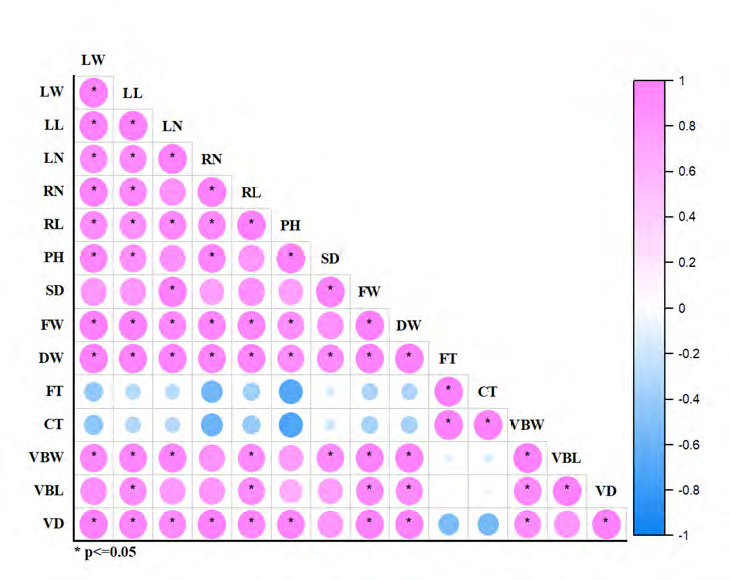
Pearson correlation analysis between 14 characteristics of Kang Kog pumpkins grown under different salinity stress levels. *Notes.* LW = Leaf width; LN = Leaf number/plant; LL = Leaf length; RN = Root number/plant; RL = Root length; PH = Plant height; SD = Stem diameter; FT = Fibre strand thickness; CT = Cuticle thickness; VBW = Vascular bundle – width; VBL = Vascular bundle – length; VD = Vessel diameter.

**Figure 4:**
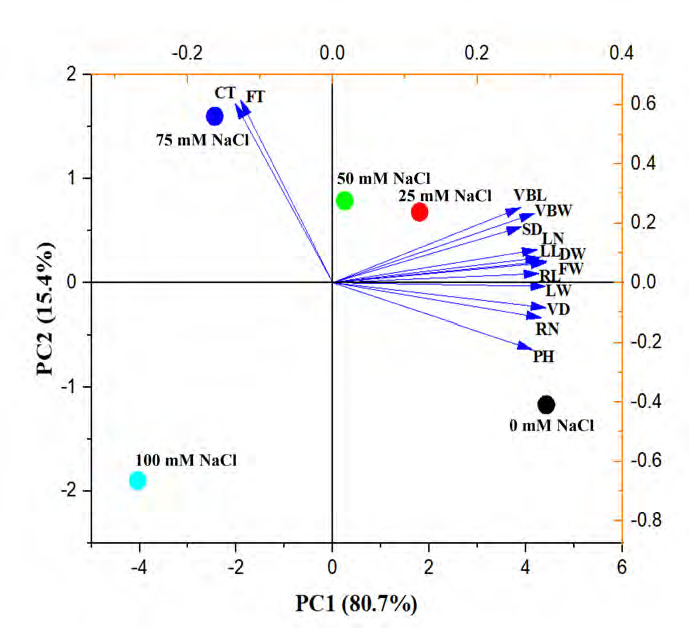
PCA biplot illustrating the relationships between the parameters and how they affected the treatments. *Notes*. LW = Leaf width; LN = Leaf number/plant; LL = Leaf length; RN = Root number/plant; RL = Root length; PH = Plant height; SD = Stem diameter; FT = Fibre strand thickness; CT = Cuticle thickness; VBW = Vascular bundle – width; VBL = Vascular bundle – length; VD = Vessel diameter.

Correlation analysis between the nine growth characteristics and five anatomical traits revealed that fiber strand thickness and cuticle thickness exhibited a negative correlation (Fig. 3). Principal Component Analysis (PCA) was used to analyse the relationship between various factors and the stress response on the growth, physiological and anatomical alterations of Kang Kog pumpkin seedlings. A PCA biplot was generated to show the correlation coefficients. The data variations were explained by fourteen components. The biplot, produced using PC1 and PC2, clarified the data correlation at 96.10%. PC1 exhibited 80.70% of the correlation, while PC2 showed 15.40% of the correlation (Fig. 4). Vascular bundle – length (VBL), vascular bundle – width (VBW), stem diameter (SD), leaf number/plant (LN), leaf length (LL), fresh weight (FW), dry weight (DW), and root length (RL) were positively correlated and situated on the positive side of PC1, while leaf width (LW), vessel diameter (VD), root number (RN), and plant height (PH) were located on the negative side of PC1. The clusters of 75 mM and 100 mM NaCl treatments were plotted in the two left-side quadrants due to the positive correlation between cuticle thickness (CT) and fiber strand thickness (FT).

## DISCUSSION

Results showed that salinity stress impacted Kang Kog pumpkin growth, similar to other plant species such as melon, sorghum, water dropwort and watermelon (Akrami & Arzani 2019; Zhang *et al*. 2020; Kumar *et al*. 2021; Shin *et al.* 2021). Salinity stress causes plants to develop more slowly, with reduced leaf size and root elongation (Neves-Piestun & Bernstein 2001). Plant roots in soil or a solution containing high salt concentration incur altered water potential (ψ). Salinity conditions induce lower water potential, causing the water in the plant roots to diffuse out, thereby impacting the absorption of water and nutrients, especially potassium, which is necessary for plant growth (Horie *et al*. 2012; Park *et al*. 2016; Zhao *et al*. 2021; Lu & Fricke 2023). When plants are exposed to long-term salinity stress, high amounts of NaCl accumulate within leaf cells, which affects biochemical processes, especially photosynthesis (Carillo *et al*. 2011; Isayenkov & Maathuis 2019). The plants had higher transpiration rates because of high salt accumulation in the leaves (Tian *et al*. 2020). This caused the leaves to turn yellow, wither and eventually die.

Pumpkins are considered to be glycophytes as plants that are sensitive to salinity but have a limited tolerance to salinity stress (Zhu *et al*. 2008) because of their deep root structure which promotes groundwater absorption (Bischoff 2018). Pumpkin sensitivity to salinity levels depends on the species or variety. Previous studies found that *C. moschata* was more sensitive to salinity stress than *C. maxima* because *C. maxima* had higher levels of ABA accumulation in its leaves, which limited water loss by rapidly closing the stomata in the early stages of salinity stress (Niu *et al*. 2018). Our results concurred with previous reports on *Cucurbita* species grown under salinity stress, with decreased growth, small pale-yellow leaves, short, dwarfed stems and a decrease in both fresh and dry weight (Tang *et al*. 2018). Increases in the antioxidant enzymes superoxide dismutase (SOD), catalase (CAT), glutathione reductase (GR) and ascorbate peroxidase (APX) caused lipid peroxidation (Sevengor *et al*. 2011). This result supported our earlier research, which suggested that ‘Butternut’ pumpkin (Taratima *et al*. 2022), including the ‘Laikaotok’ pumpkin – a local cultivar in Thailand (Taratima *et al*. 2023), was negatively impacted by saline stress. However, this effect depends on the different salinity levels.

The results from this study proved that the photosynthetic efficiency of Kang Kog pumpkin decreased after cultivation under salinity stress. Following salinity treatment in this investigation, the total chlorophyll and chlorophyll *a* contents were significantly lower than the control, while the leaf green intensity (SPAD unit), chlorophyll *b* content and chlorophyll fluorescence measured under both dark-adapted (Fv/Fm units) and light condition (Fv’/Fm’ units) slightly decreased but were not statistically different from the control. This result concurred with numerous reports that salinity stress reduced the efficiency of photosynthesis in many plant species such as sweet sorghum varieties (Baiseitova *et al*. 2018), common bean (*Phaseolus vulgaris* L.) (Taïbi *et al*. 2016), *Kalidium foliatum* (Gong *et al*. 2018) and *Reaumuria soongorica* (Yan *et al*. 2022). Plants that experience salinity stress exhibit lower levels of photosynthetic pigments, such as carotenoids and total chlorophyll, leading to chlorosis, which manifests as pale leaves, and a decrease in photosynthetic activity (Trifunović-Momčilov *et al*. 2021). This is caused by the accelerated breakdown or inhibition of chlorophyll synthesis (Santos 2004). Moreover, salinity stress affects the photosynthetic process because when the leaves lose water, the stomata close, resulting in reduced carbon dioxide fixation (Xu & Zhou 2008; Takagi *et al*. 2009; De Oliveira *et al*. 2013; Hnilickova *et al*. 2017). Changes in the cell membrane properties also limit carbon dioxide movement through the cells, while reduced potassium absorption impacts the photosystem (Parihar *et al*. 2015; Wang *et al*. 2017), depending on the efficiency of the light reaction (Qu *et al*. 2012). Therefore, when plants are exposed to salinity stress, it affects the activity of photosystem II (PSII) (Allakhverdiev *et al*. 2000), causing damage and reducing the photochemical reaction (ΦPSII) (Misra *et al*. 2001; Srivastava & Sharma 2021). Salinity stress levels are typically determined by measuring chlorophyll fluorescence yield, which is often reduced under salt stress (Kalaji *et al*. 2011; Harizanova & Koleva-Valkova 2019; ALKahtani *et al*. 2020; Hosseini *et al*. 2023). The chlorophyll fluorescence of Kang Kog pumpkin in this study slightly decreased after cultivation under different salinity levels but the values obtained were not significantly different from the control. Chlorophyll fluorescence analysed in this study corresponded to chlorophyll *b* content that was not significantly different between the treatments and the control. This result agreed with Gong *et al*. (2018) who found that salinity stress did not affect chlorophyll *a* content but impacted total chlorophyll and chlorophyll *a* of *Kalidium foliatum*. Plants exposed to salinity stress generate free radicals to destroy the thylakoid membrane (Yamane *et al*. 2004). This causes chlorophyll to become damaged and lose its properties, turning it into a colorless substance. This process is known as photobleaching of chlorophyll (Lingvay *et al*. 2020).

In addition to affecting growth, physiological and biochemical characteristics, salinity stress also impacts plant anatomy, particularly after salinity stress treatment, vascular bundle size and vessel diameter of all treatments group in this investigation decreased compared to the control group. This result concurred with previous studies in barley seedlings (*Hordeum vulgare* L.) (Atabayeva *et al*. 2013), *Gazania harlequin* L. (Younis *et al*. 2014), *Astragalus gombiformis* (Boughalleb *et al*. 2017), *Salicornia freitagii* (Akcin *et al*. 2017), and tomato (*Solanum lycopersicum* Mill.) (Alsafari *et al*. 2019). Vascular bundles and vessel diameters respond to salinity stress by shrinking to reduce water uptake (Hampson & Simpson 1990). When plants are subjected to salt stress, they absorb ions such as Na^+^ and Cl^−^, which may be detrimental to their cells. Therefore, reduced vascular bundle and vessel size involve the controlled absorption of Na^+^ and/or Cl^−^, which is the main process influencing salt tolerance (Singh *et al*. 2017). In this investigation, two notable anatomical characteristics—fibre strand thickness and cuticle thickness— were identified as important indicators for evaluating salt-tolerant pumpkin. Our results showed that these two anatomical traits increased after salinity treatment, particularly in the 75 mM NaCl treatment. This result aligns with previous studies on certain plant species after salinity treatment, such as soybean (Dolatabadian *et al*. 2011) and ‘Butternut’ pumpkin (Taratima *et al*. 2022). However, the fiber strands discussed in this study refer to sclerenchyma fibres, not cotton fibres or other elongated epidermal cells (hairs). Growing cotton plants under salt stress conditions results in lower-quality fibre (Chaudhary *et al*. 2024).

Generally, fibre plant cells have secondary cell walls composed of cellulose, hemicellulose, lignin, suberin, pectin and other polysaccharides (Zhao *et al*. 2021). Under salinity stress, cell wall synthesis is controlled by intricate transcriptional systems incorporating phytohormones, especially ABA (Zhong *et al*. 2010; Endler & Persson 2011; Wang *et al*. 2020). Previous reports revealed that plant response to salinity stress is greatly influenced by the biological functions of cellulose, lignin and other polysaccharides present in the cell walls (Endler & Persson 2011; Liu *et al*. 2018). Plant cells exposed to salt stress adapt by building up lignin and strengthening their cell walls. This phenomenon proves that lignin is crucial for plant adaptation under salt stress (Zhao *et al*. 2021). The thickening of the cuticle layer is an important mechanism that helps plants to reduce cell water loss. Under severe salinity stress, plants lose water until their cells shrivel up and die (Kosma & Jenks 2007; Samuels *et al*. 2008).

## CONCLUSION

Pumpkins have a wide range of cultivars that develop differently under salt stress. This study showed that the growth of Kang Kog pumpkin was impacted by moderate and high salinity stress conditions but the plant was able to adapt and grow under slight salinity stress. Chlorophyll *b* and chlorophyll fluorescence of the treated groups were not significantly different from the control group, despite the reduced growth, demonstrating the physiological adaptation of this pumpkin cultivar. Characteristics indicating anatomical adaptation under salt stress conditions included fibre strand thickness and cuticle thickness. These traits can be used to evaluate the salinity tolerance of pumpkins or other related plant species. The knowledge gained from this research can be used to support the planting programme of local cultivar pumpkins in various saline soil areas in Thailand. Nevertheless, this study was limited to the seedling stage of hydroponic systems. To more definitively confirm the findings, additional research is needed in the future extending to harvesting stage, including planting in soil with varying salinity levels.

## Figures and Tables

**Table 1:** Growth, physiological and anatomical characteristics of Kang Kog pumpkin after four weeks of different salinity stress treatments.

Characteristic	NaCl concentration (mM) ± SD)				

	0	25	50	75	100
Leaf number/plant	9.60 ± 2.3^a^	7.40 ± 0.5^b^	6.40 ± 0.5^b^	4.00 ± 1.0^c^	1.20 ± 0.4^d^
Leaf width (cm)	13.42 ± 1.8^a^	10.50 ± 1.0^b^	8.12 ± 1.5^c^	6.70 ± 1.5^d^	4.70 ± 0.3^e^
Leaf length (cm)	11.20 ± 1.5^a^	9.46 ± 0.9^b^	7.04 ± 1.3^c^	6.70 ± 1.5^c^	4.00 ± 0.1^d^
Root number/plant	27.40 ± 3.1^a^	21.60 ± 4.1^b^	13.60 ± 2.9^c^	6.60 ± 3.1^d^	6.00 ± 1.7^d^
Root length (cm)	21.36 ± 1.5^a^	20.14 ± 1.4^a^	19.20 ± 3.4^a^	13.64 ± 4.5^b^	12.98 ± 1.9^b^
Plant height (cm)	34.89 ± 5.7^a^	20.22 ± 0.8^b^	16.52 ± 3.8^b^	11.46 ± 1.4^c^	10.50 ± 0.6^c^
Stem diameter (cm)	0.78 ± 0.1^a^	0.62 ± 0.0^b^	0.73 ± 0.0^a^	0.57 ± 0.0^b^	0.39 ± 0.1^c^
Fresh weight (g)	27.24 ± 1.2^a^	22.39 ± 1.4^b^	15.46 ± 1.2^c^	9.31 ± 1.4^d^	2.50 ± 0.3^e^
Dry weight (g)	3.42 ± 0.2^a^	2.67 ± 0.3^b^	2.15 ± 0.2^c^	1.30 ± 0.1^d^	0.46 ± 0.0^e^
Total Ch (mg/g FW)	4.19 ± 0.6^a^	3.61 ± 0.6^ab^	2.97 ± 0.9^b^	2.99 ± 0.7^b^	ns
Ch a (mg/g FW)	2.57 ± 0.4^a^	2.11 ± 0.3^ab^	1.74 ± 0.3^bc^	1.39 ± 0.1^c^	ns
Ch b (mg/g FW)	1.61 ± 0.2^a^	1.51 ± 0.1^a^	1.23 ± 0.2^a^	1.60 ± 0.3^a^	ns
SPAD (units)	40.98 ± 7.6^a^	35.07 ± 4.7^ab^	31.50 ± 3.5^b^	17.68 ± 5.3^c^	ns
Ch flu. Fv’/Fm’	0.80 ± 0.0^a^	0.81 ± 0.0^a^	0.79 ± 0.0^a^	0.76 ± 0.0^a^	ns
Ch flu. Fv/Fm	0.73 ± 0.0^a^	0.72 ± 0.0^a^	0.71 ± 0.0^a^	0.71 ± 0.0^a^	ns
Fibre strand thickness (μm)	58.18 ± 11.5^b^	67.03 ± 5.6^ab^	68.41 ± 6.5^ab^	73.93 ± 7.6^a^	61.91 ± 6.7^b^
Cuticle thickness (μm)	2.62 ± 0.8^c^	3.96 ± 1.3^ab^	4.09 ± 0.8^ab^	5.06 ± 1.1^a^	3.23 ± 0.4^bc^
Vas. Bun. – width (μm)	603.07 ± 100.1^a^	534.71 ± 78.9^b^	478.84 ± 84.2^bc^	388.26 ± 46.3^c^	167.61 ± 35.8^d^
Vas. Bun. – length (μm)	749.83 ± 108.9^ab^	877.90 ± 65.6^a^	597.73 ± 108.3^bc^	491.48 ± 78.4^c^	228.63 ± 42.8^d^
Vessel diameter (μm)	130.41 ± 9.1^a^	96.58 ± 10.1^b^	74.19 ± 11.3^c^	42.70 ± 4.6^d^	35.99 ± 2.0^d^

*Notes*. Mean ± SD values followed by different superscripts in the same row are significantly different according to ANOVA and Duncan’s Multiple Range Test (*p* < 0.05). Ch = Chlorophyll; Ch flu. = Chlorophyll fluorescence; Vas. Bun. = Vascular bundle; ns = non-scorable.
